# Excessive Cytosolic DNA Fragments as a Potential Trigger of Graves’ Disease: An Encrypted Message Sent by Animal Models

**DOI:** 10.3389/fendo.2016.00144

**Published:** 2016-11-14

**Authors:** Yuqian Luo, Aya Yoshihara, Kenzaburo Oda, Yuko Ishido, Koichi Suzuki

**Affiliations:** ^1^Department of Clinical Laboratory Science, Faculty of Medical Technology, Teikyo University, Tokyo, Japan; ^2^Department of Education Planning and Development, Faculty of Medicine, Toho University, Tokyo, Japan; ^3^Department of Internal Medicine, Division of Diabetes, Metabolism and Endocrinology, Toho University, Tokyo, Japan

**Keywords:** Graves’ disease, thyroid-stimulating hormone receptor, major histocompatibility complex class II, genomic DNA, experimental animal models

## Abstract

Graves’ hyperthyroidism is caused by autoantibodies directed against the thyroid-stimulating hormone receptor (TSHR) that mimic the action of TSH. The establishment of Graves’ hyperthyroidism in experimental animals has proven to be an important approach to dissect the mechanisms of self-tolerance breakdown that lead to the production of thyroid-stimulating TSHR autoantibodies (TSAbs). “Shimojo’s model” was the first successful Graves’ animal model, wherein immunization with fibroblasts cells expressing TSHR and a major histocompatibility complex (MHC) class II molecule, but not either alone, induced TSAb production in AKR/N (H-2^k^) mice. This model highlights the importance of coincident MHC class II expression on TSHR-expressing cells in the development of Graves’ hyperthyroidism. These data are also in agreement with the observation that Graves’ thyrocytes often aberrantly express MHC class II antigens *via* mechanisms that remain unclear. Our group demonstrated that cytosolic self-genomic DNA fragments derived from sterile injured cells can induce aberrant MHC class II expression and production of multiple inflammatory cytokines and chemokines in thyrocytes *in vitro*, suggesting that severe cell injury may initiate immune responses in a way that is relevant to thyroid autoimmunity mediated by cytosolic DNA signaling. Furthermore, more recent successful Graves’ animal models were primarily established by immunizing mice with TSHR-expressing plasmids or adenovirus. In these models, double-stranded DNA vaccine contents presumably exert similar immune-activating effect in cells at inoculation sites and thus might pave the way toward successful Graves’ animal models. This review focuses on evidence suggesting that cell injury-derived self-DNA fragments could act as Graves’ disease triggers.

## Introduction

Graves’ disease is a unique human autoimmune disease that involves stimulating autoantibodies directed toward thyroid-stimulating hormone receptors (TSHRs) on the surface of thyroid epithelial cells. This disease occurs in approximately 3% of females and 0.5% of males in the general population (1). Unlike autoantibodies to thyroglobulin (Tg) or thyroid peroxidase (TPO), thyroid-stimulating TSHR autoantibodies are not just a marker of Graves’ disease but are also held directly responsible for the hyperthyroidism that occurs in most of the patients. Moreover, evidence suggests that only TSHR is the primary autoantigen of Graves’ disease, whereas immune responses to other thyroid antigens (e.g., Tg and TPO) simply reflect concomitant thyroiditis.

TSHR autoantibody was first discovered in a search for thyroid-stimulating activity in the serum of Graves’ disease patients, which was known to stimulate radioiodine release from pre-labeled guinea pig thyroids for a much longer time period than did pituitary TSH treatment (2). This prolonged stimulating activity present in the IgG fraction of Graves’ patient serum could compete with TSH for TSHR occupancy, which implies the presence of TSHR antibodies that act as TSHR agonists (3). Thus, in most Graves’ disease patients, circulating antibodies that have TSH-like activity continuously stimulate the thyroid. This continuous stimulation results in an enlarged thyroid known as goiter, and these patients have increased iodine uptake and overproduction of thyroid hormone (TH). Typical blood tests for Graves’ disease patients show elevated T3 and T4 levels, as well as low TSH (as a result of negative feedback loop) levels (4).

TSHR antibodies (TRAbs) in serum from Graves’ disease patients can now be clinically evaluated by non-radioactive third generation assay, in which the autoantibodies inhibit binding of a biotin-labeled human monoclonal thyroid-stimulating antibody M22 to TSHR-coated ELISA plate wells (5). However, TRAbs may or may not initiate a TSH-like intracellular signal. TRAbs that induce a strong TSH-like stimulatory signal are referred to as TSHR-stimulating antibodies/immunoglobulin (TSAbs/TSI), which is the immunological hallmark of Graves’ disease. Meanwhile, TRAbs that induce weak or no stimulatory signals are referred to as TSHR-blocking antibodies (TBAbs). The TSAb activity of TRAbs is usually evaluated by their capacity to induce cAMP production in TSHR-expressing cells (6). TSAbs and TBAbs can sometimes coexist in the serum of an individual patient and may change over time. The clinical status of a patient who has both TSAbs and TBAbs presumably depends on the relative concentration and affinity of the predominant antibody type. A shift from TSAbs to TBAbs may occur during spontaneous or treatment-induced remission of Graves’ disease that may lead to the subsequent development of hypothyroidism (7). In addition to TRAbs, TPO and/or Tg antibodies are detectable in 25–75% of Graves’ disease patients, which is consistent with the lymphocytic infiltration seen in Graves’ thyroids and is typically less extensive than that seen for Hashimoto’s disease.

Although its characteristic hyperthyroidism symptoms and the availability of sensitive laboratory tests may make the diagnosis of Graves’ disease straightforward, the lack of an understanding of the pathogenic mechanisms of this disease has impeded the development of cures. In Graves’ disease, immune tolerance toward self-antigen TSHR is obviously dysfunctional, such that, from a classical point of view, endogenous TSHR processed in the cytosol of thyrocytes gives rise to peptides for human leukocyte antigen (HLA) class I presentation to CD8^+^ T cells. Alternatively, TSHR may be engulfed by antigen-presenting cells (APCs, typically macrophages, dendritic cells, and B cells) where it is digested in the lysosomes and destined for HLA class II presentation to CD4^+^ T cells. In order to dissect Graves’ disease pathogenesis, tremendous efforts have been made to develop experimental Graves’ animal models that have indeed provided invaluable insights for understanding the reasons behind the breakdown of self-tolerance.

## Animal Models of Graves’ Disease

**Figure 1:**
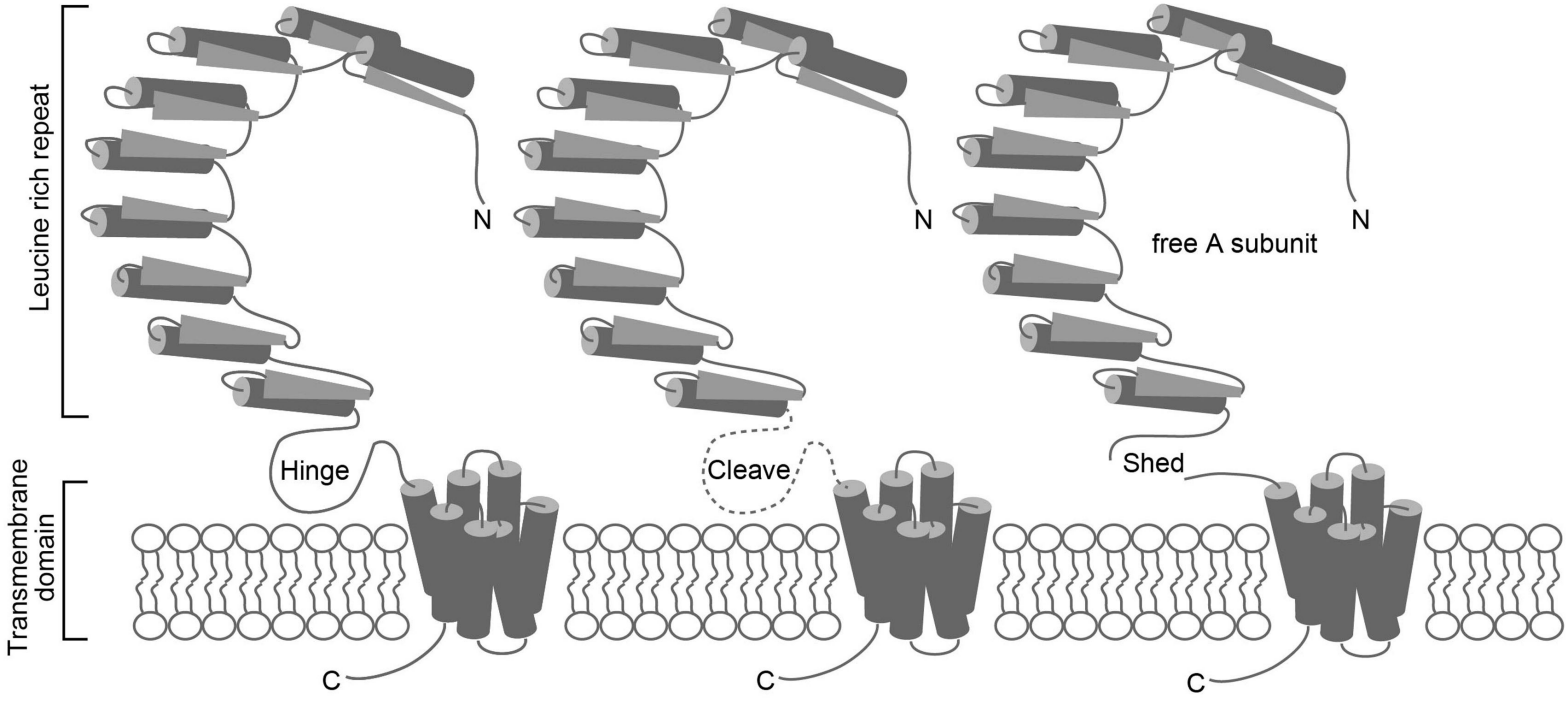
**Schematic representations of the TSHR protein, intramolecular cleavage, and A subunit shedding**. TSHR consists of an A subunit that has a large extracellular horseshoe-shaped leucine-rich repeat region (LRR) known as the ectodomain, and a B subunit with seven transmembrane regions (left). TSHR undergoes intramolecular cleavage at a hinge-like single-chain polypeptide on the cell surface that connects the A and B subunits (middle). The cleaved receptor is susceptible to loss of the A subunit by shedding (right).

Autoimmune thyroiditis occurs spontaneously in several animal species (8–10); however, Graves’ disease develops spontaneously only in the humans. Conventional animal models of autoimmune thyroiditis that is produced by immunizing animals with Tg or TPO protein have long been available (11). After human TSHR was cloned, similar attempts were made to induce Graves’ disease by immunizing animals with human TSHR that was expressed either in a baculovirus expression system or in insect cells (12–16), or purified from cloned human thyroid cells (GEJ) (17). The TSHR is a member of the G protein-coupled receptor superfamily and is coupled with the Gs protein that activates the cAMP-dependent pathway (18). TSHR consists of a short cytoplasmic C-terminal tail, seven transmembrane regions, and a large extracellular horseshoe-shaped leucine-rich repeat region (LRR) known as the ectodomain (Figure 1) (18). TSHR reportedly undergoes intramolecular cleavage at some portion of the single-chain polypeptide on the cell surface to form two subunits, such as A and B, which are linked by a hinge of disulfide bonds (19). The extracellular ectodomain (A subunit) of the cleaved receptor is also susceptible to loss by shedding (20, 21) (Figure 1). In addition, epitopes for TSAb, but not TBAb, are partially obstructed in wild-type TSHR by the plasma membrane, LRR, or TSHR dimerization. However, the TSAb epitope on the soluble A subunit that is shed from surface TSHR is freely accessible (22). These observations suggest that the shed A subunit, rather than the cell surface full-length TSHR, may be responsible for initiating or amplifying the autoimmune response to the TSHR that in turn leads to Graves’ hyperthyroidism. To provide evidence to support this concept, various TSHR ectodomain preparations instead of the full-length TSHR were more frequently used to immunize animals for the development of Graves’ disease animal models (12–16). Although serum antibodies and murine monoclonal antibodies against hTSHR were generated in these immunized animals, antibodies with TSAb activity were absent, despite the use of various TSHR preparations with different adjuvants in a variety of mouse strains. The animals did not display increased serum TH, goiter, or thyrocytes hypertrophy either. Human TSHR was thought not to be an authentic autoantigen and thus was unsuitable for inducing autoantibodies in mice. However, even immunization of mice with purified murine TSHR ectodomain expressed in insect cells with an adjuvant failed to induce hyperthyroidism (23).

Purified TSHR peptides expressed in bacteria or insect cells might lack a functional conformation that is needed to induce TSAb in animal models by conventional immunization approaches. To overcome this obstacle, later models used immunization approaches that involved *in vivo* expression of THSR. In these models, animals are injected with transfected cells stably expressing hTSHR, or with plasmids or adenovirus for transient hTSHR expression. The first authentic animal model of Graves’ disease known as “Shimojo’s model” was generated by intraperitoneal immunization of female AKR/N (H-2^k^) mice with murine fibroblasts that stably expressed full-length hTSHR and a major histocompatibility complex (MHC) class II molecule (24). The use of fibroblasts that express both TSHR and also MHC class II molecules was based on the observation of aberrant expression of MHC class II molecules on thyrocytes from patients with autoimmune thyroid diseases (AITD), including Graves’ disease (25). Such observations raised the possibility that TSHR might be presented to immune system by MHC class II-expressing thyrocytes that would break down normal immune tolerance (26). AKR/N (H-2^k^) mice were used for these models, as they have a homologous MHC class II I-A molecule that matches the expressed MHC class II molecule on fibroblasts (24). Approximately 20% of immunized mice produced TSAbs and showed increased T4 and T3 levels, as well as goiter with minimal lymphocyte infiltration (24). Intriguingly, immunizing mice with fibroblasts transfected with either TSHR or MHC class II alone did not induce Graves’ hyperthyroidism (24), indicating that aberrant expression of MHC class II molecules on cells expressing a native form of the TSHR can induce TSAb production. Shimojo’s group later tried the same approach with several different mouse strains that shared the H-2^k^ haplotype but had different genetic backgrounds and found that, unlike AKR/N mice, C3H/He mice generated TBAbs even in the absence of MHC class II expression (27). However, simultaneous MHC class II expression was needed for TSAb production and development of hyperthyroidism (27). These results suggest that some genetic backgrounds are more susceptible to the induction of TRAbs, while for the development of functional TRAbs, aberrant MHC class II expression is necessary. Since “Shimojo’s model” was first described, many other independent groups have tried to reproduce and improve this model (28–31). For example, intraperitoneal immunization with TSHR-expressing M12 (B cells) induced Graves’ hyperthyroidism with TSAbs in 100% of immunized BALB/c mice (29, 30). Additionally, intraperitoneal immunization with hTSHR-expressing Chinese hamster ovary (CHO) cells induced Graves’ hyperthyroidism in 20% of immunized female Chinese hamsters (28). A noteworthy detail of this study is that the CHO cells used in this immunization approach constitutively expressed MHC class II mRNA as demonstrated by RT-PCR (28). In addition to *in vivo* expression of TSHR, the approaches in these studies shared immunization protocols that included cells that were either stably transfected with MHC class II-encoding cDNA (24, 27, 31, 32) or constitutively expressed MHC class II (28–30). This common feature also leads to a limitation wherein these models are only applicable to syngeneic animals that share the same MHC class II haplotype as the cells used for immunization. Thus, both the success and limitation seem to indicate an important role for aberrant MHC class II expression in the induction of TSAbs and development of Graves’ hyperthyroidism.

In order to overcome the strain limitation in Shimojo’s approach, novel immunization approaches that relied on intramuscular injection of plasmid vectors encoding TSHR to induce transient TSHR expression by myoblasts at the injection site (33), a method known as “naked” DNA vaccination, were developed. Intramuscular TSHR DNA vaccination was first tried in female BALB/c mice, which gave rise to TRAbs with TBAb activity in 10 of the 14 immunized mice, whereas weak TSAb activity was detectable in only 1 mouse (34). Severe intrathyroidal lymphocyte infiltration was observed in all the immunized mice, although none developed Graves’ hyperthyroidism (34). Thus, these initial attempts using DNA vaccination, despite their ability to generate TRAbs that recognized native TSHR, poorly fulfilled their initial promise of producing TSAbs. The TSHR DNA vaccination approaches were then modified, and different mouse strains were used. In outbred NMRI mice, intramuscular DNA vaccination produced TSAb and hyperthyroidism in 15% of females and 3% of males (35). For BALB/c mice, extensive lymphocyte infiltration was observed in most of the immunized outbred NMRI mice (35). Moreover, intradermal injection of TSHR DNA induced TSAb and hyperthyroidism in inbred female BALB/c mice at an incidence of 27% (36). Indeed, skin could be a better anatomical site for DNA vaccination since the skin is enriched in dendritic cells (Langerhans’ cells) that phagocytize and present antigens (36).

Thyroid-stimulating hormone receptor endogenously expressed by vaccination is presumably presented preferentially through the MHC class I antigen pathway; however, the involvement of MHC class II presentation may also be necessary for optimal T cell signaling during TSAb production, as implied in “Shimojo’s model.” To test this hypothesis, Pichurin et al. constructed a chimeric plasmid that encodes TSHR and the lysosome-associated membrane protein (LAMP)-1, which has a sorting signal that can direct TSHR into lysosomes and, consequently, into the MHC class II presentation pathway. A chimeric TSHR–LAMP-1 plasmid was tested for its efficacy in intramuscular DNA vaccination. Remarkably, TSAb and hyperthyroidism were induced in approximately 20% of female BALA/c mice presumably through hijacking of the TSHR to the MHC class II presentation pathway (37). In contrast, no mice of the same strain injected with wild-type TSHR DNA vaccine showed TSAbs and hyperthyroidism (32). These results indicate that engaging MHC class II presentation facilitates the generation of TSAbs. Additionally, 30% of murine MHC class II knockout HLA-DR3 transgenic NOD mice vaccinated with TSHR DNA showed TSAb induction and Graves’ hyperthyroidism (38). These observations, together with the results for BALB/c and outbred mice, indicate that, although theoretically DNA vaccination can be performed in any mouse strain, genetic background remains a decisive factor in DNA vaccination outcomes. Despite the various mouse strains tested and modifications to the immunization protocols, disease incidence induced by DNA vaccination was low (0–30%) (32, 35–42). This low success rate can presumably be attributed to the relatively low TSHR expression efficiency afforded by plasmid vectors.

Substituting adenovirus for plasmid as the vector that encodes THSR cDNA has generally increased the incidence of Graves’ hyperthyroidism in mice. In the original report, intramuscular adenovirus (ad)-TSHR immunization induced TSAb and hyperthyroidism symptoms in 55 and 33% of female and male BALB/c mice, respectively. C57BL/6 mice were less susceptible in that only 25% of the females developed hyperthyroidism after the ad-TSHR immunization. Meanwhile, CBA/J, DBA/1J, and SJL/J mice were completely resistant to ad-TSHR-induced hyperthyroidism (43). By adapting the adenovirus vector to express the TSHR A subunit instead of full-length TSHR, the incidence of induced Graves’ disease was increased to approximately 65–80% in female BALB/c mice (44, 45). Although intramuscular injection of plasmid-TSHR was less effective than ad-TSHR for inducing Graves’ disease in mice, the adoption of intramuscular electroporation for plasmid-TSHR genetic immunization has recently achieved considerable improvement in disease induction as manifested by *in vivo* hTSHR expression and significantly increased disease incidence (46, 47). Surprisingly, TSAbs persisted for more than 8 months after the final electroporation immunization (46), which is in contrast to the transient hyperthyroidism induced by intramuscular immunization wherein TSAb activity often began to decline much earlier or even completely disappeared (48). Another recently reported model of long-term Graves’ disease was established by prolonged intramuscular immunization with the ad-TSHR A subunit in female BALB/c mice (49). Long-term Graves’ models would be particularly useful for pharmacological analysis and for monitoring treatment response.

## Aberrant Expression of MHC Class II on Thyrocytes

By immunizing mice with fibroblasts transfected with both hTSHR and a MHC class II molecule, but not by either alone, Shimojo et al. was the first to successfully generate an authentic Graves’ mouse model (24). This model supported a previously proposed hypothesis that epithelial cells from organs that are highly susceptible to organ-specific autoimmunity can be induced to express MHC class II antigens and in turn present antigens to T cells (26). Unlike MHC class I, which is expressed in many types of nucleated cells, MHC class II expression is restricted to professional APCs, such as macrophages, dendritic cells, and B cells. However, aberrant MHC class II expression on thyroid epithelial cells is frequently seen in thyroid autoimmune diseases. Hanafusa et al. used immunofluorescence staining to demonstrate aberrant HLA-DR expression in discrete groups of follicles in 20 of the 26 thyroids from patients with Graves’ disease, whereas none of 11 specimens from normal thyroids did (25). Similarly, Jansson et al. reported that HLA-DR-positive thyrocytes were observed in 9 of the 11 specimens of Graves’ thyroids by immunohistochemical staining (50). In addition to thyroid autoimmune diseases, aberrant HLA-DR expression on epithelial cells has also been noted in other organ-specific autoimmune diseases, including type I diabetic insulitis (51). Although MHC class II expression is not constitutive, immune mediators can induce MCH class II production in epithelial cells. Interferon (INF)-γ, known as the most prominent MHC class II stimulator, can induce MHC class II on thyrocytes both *in vitro* and *in vivo* (52–54). INF-γ is predominantly produced by lymphocytes as part of innate immune responses (55). Thus, whether aberrant MHC class II expression on Graves’ thyrocytes is secondary to coincident lymphocyte infiltration has been wondered. However, so far, the spatial relationship between HLA-DR-positive thyrocytes and lymphocyte foci in Graves’ thyroids remains obscure due to conflicting observations (25, 50).

Besides the undetermined trigger for aberrant MHC class II expression on thyrocytes, little is known about MHC class II antigen presentation in these cells. In contrast to professional APCs, thyrocytes do not have naturally well-adapted machinery for either phagocytosis or antigen processing and presentation, and they do not migrate to lymphoid organs. Nevertheless, there are several lines of evidence to suggest that MHC class II-positive thyrocytes may present peptides to and directly interact with homologous T cells. Induced HLA-DR-positive thyrocytes could promote proliferation of autologous T cells *in vitro*, a phenomenon that does not occur in the absence of HLA-DR expression and is inhibited by HLA-DR monoclonal antibodies (56). Moreover, in a study of 18 Graves’ disease patients, expression of HLA-DR antigens on thyrocytes after primary culture in the absence of INFγ was seen in 12 patients, and this expression induced proliferation of autologous T cells derived from both thyroids and peripheral blood (53, 57). T lymphoblast generation was also observed after culturing normal spleen lymphocytes on monolayers of syngeneic thyrocytes for 3 days. Intriguingly, only these T lymphoblasts that had been sensitized on thyrocytes were specifically labeled with fluorescein-conjugated Tg (58). Additionally, primary Graves’ thyrocytes were shown to possess phagocytic activity that was enhanced by interleukin-2 and INF-γ and inhibited by antithyroid drugs and steroid medications (59). HLA-DR-positive thyrocytes could present to cloned human T cells an influenza-specific peptide, but not an intact flu virus, and this reaction was blocked by HLA-DR antibodies (60).

Another intriguing question is how aberrantly expressed MHC class II on thyrocytes contributes to breaking self-tolerance. Pichurin et al. demonstrated that hijacking endogenously expressed TSHR into the MHC class II presentation pathway by using a chimeric plasmid encoding both TSHR and the lysosome-directing molecule LAMP was significantly more effective for inducing Graves’ hyperthyroidism in BALB/c mice than the use of plasmids encoding wild-type TSHR (37). This finding indicates that the more endogenous antigens entered the MHC class II pathway, the more TSAbs would be generated. Traditionally, immunologists held that MHC class I and II were restricted to the cytosol and endosomes/lysosomes, respectively, for surveying distinct subcellular domains for ligands. However, alternative pathways for delivering exogenous antigens to MHC class I have been characterized and are known as cross-presentation (61). On the other hand, the observation that a large proportion of peptides purified from MHC class II are derived from cytosolic self-proteins (e.g., metabolic enzymes, cytoskeletal proteins, and tumor antigens) indicates that MHC class II may also present endogenous peptides for CD4^+^ T cell recognition, which has potential relevance to autoimmunity and tumor immunity (62, 63). Endogenously expressed viral proteins were shown to be lysed by MHC class II-restricted virus-specific CD4^+^ T cells (64), indicating that endogenously expressed proteins can be presented by the MHC class II pathway for CD4^+^ T cell recognition. Moreover, endogenous antigen presentation by MHC class II could occur through both autophagy-dependent [reviewed in Ref. (65)] and autophagy-independent pathways [reviewed in Ref. (66)]. Meanwhile, the observation that endogenously expressed TSHR A subunits are in general more efficient than non-cleaving TSHR and wild-type TSHR for inducing TSAb and Graves’ hyperthyroidism in animal models (45) depicts another possible scenario, in which the shed TSHR A subunit might be internalized by MHC class II-positive thyrocytes and presented through the conventional lysosome/endosome-MHC class II pathway. It is possible that the pathways by which MHC molecules acquire peptides have a significant impact on the generation of peptide diversity that will be ultimately recognized by the T cells and give rise to diverse antibodies.

## APC Adaptation in Thyrocytes Stimulated by Cytosolic DNA

Major histocompatibility complex class II expression on the transferred fibroblasts was a key factor for the generation of TSAb in “Shimojo’s model” (24). In genetic immunization models, vaccines usually consist of TSHR-expressing vectors (plasmids or adenovirus) and sometimes with additional cytokine (such as IL-2, IL-4, and IL-12)-expressing vectors as adjuvants (36). However, none of these vaccines has ever included vectors that express MHC class II. At first glance, MHC class II expression would appear to be irrelevant in the genetic vaccine-induced Graves’ animals. Yet in 1999, Suzuki et al. surprisingly found that both MHC class I and II expression was strongly induced on the cell surface of cloned rat thyroid FRTL-5 cells after the cells were transfected with irrelevant or even empty plasmids. In order to dissect the cause for this aberrant MHC expression, they tried different transfection methods and reagents [e.g., lipofection, electroporation, and diethylaminoethyl (DEAE)-dextran] to introduce various DNA substances into FRTL-5 cells. They found that, regardless of the transfection methods and DNA origin, diverse DNA, including bacterial DNA, viral DNA, salmon sperm DNA, calf thymus DNA, self-genomic DNA, plasmid DNA, and artificially synthesized DNA (>25 bp), could induce significant MHC expression on FRTL-5 cells, whereas single-stranded DNA (ssDNA) could not (67). Later studies revealed that a classic double-stranded right-handed helix sense (B-DNA) with a native sugar–phosphate backbone is necessary for the aberrant MHC expression induced by DNA, and the effect was independent of sequence or presence of unmethylated CpG motifs (67–69). Moreover, free DNA in the extracellular medium did not induce MHC expression, indicating that this effect was likely mediated by cytosolic DNA sensors rather than cell surface receptors (67).

Besides MHC molecules, cytosolic DNA induces the expression of an array of molecules in thyrocytes that are involved in antigen-processing and -presenting pathways, such as proteasome protein LMP2, transporter associated with antigen processing (TAP), MHC II-associated invariant chain (Ii), costimulatory molecules (CD80, CD40, CD54, and CD86) (67, 70), and the production of various immune mediators, including type I IFN, TNF-α, and IL-6 (70). These observations indicate that thyrocytes were adapted to behave like APCs with activated innate immune response upon exposure to cytosolic DNA, a phenomenon that has been widely reproduced in various non-professional APC cells (such as fibroblasts, keratinocytes, epithelial cells, and endothelial cells). Cytosolic DNA similarly enhances APC activity in professional APCs (67–71). Consistent with this finding, T cells were indeed activated to a higher degree, as measured by IL-2 and IFN-γ secretion levels, when they were mixed with peptide-challenged dendritic cells containing cytosolic DNA, compared to those without cytosolic DNA, or those containing ssDNA (71). Based on these findings, it is reasonable to speculate that muscle cells at the injection sites would express MHC class II and undergo adaptations to obtain some APC-like features after immunization with either plasmids or adenovirus (both contain dsDNA structures). These APC-like adaptations occurring in DNA-stimulated cells might have played a significant role to precipitate TSAb generation in animals and may also hold a key to understand the trigger for Graves’ disease in the humans.

## Cell Injury Induces APC Adaptation *via* Cytosolic DNA Signals

Thyrocytes would likely encounter DNA in the cytosol following bacteria or virus infection that would introduce foreign DNA (72). Although abundant indirect data suggest the involvement of infecting organisms in the pathogenesis of AITD (72), there is no direct evidence for this possibility and thus the role of infection in AITD remains a subject of debate (73). In addition to foreign DNA, self-DNA that is normally sequestered within the nucleus or in the mitochondria can also enter the cytosol of phagocytes from apoptotic bodies released by dying cells *in vivo*. Phagocytes engulfing these apoptotic bodies from the extracellular medium would eliminate unnecessary DNA through DNase present in the phagolysosomes (74). DNase deficiency leads to the accumulation of large amounts of cytosolic DNA in phagocytes derived from apoptotic cells (75). The ability to remove DNA waste is indispensable for *in vivo* homeostasis. Defective clearance of self-DNA due to mutations in DNase genes is known to be associated with the development of human autoimmune diseases such as systemic lupus erythematosus (SLE) (76, 77), indicating that excessive self-DNA can be a potential trigger for breaking self-tolerance.

Even with normal DNase function, severe cellular injuries, depending on the magnitude, can result in a large amount of DNA waste that outpaces the intrinsic clearance rate of the phagocytes and, thus, would inevitably lead to rapid cytosolic DNA accumulation. In order to demonstrate whether sterile cell injury would cause cytosolic DNA accumulation that is sufficient to induce an APC-like adaptation and stimulate an innate immune response in normal thyrocytes, Kawashima et al. applied electric pulses of increasing intensity to cultured thyrocytes and found that the amount of cytosolic DNA increased in a current intensity-dependent manner and correlated with significantly increased expression of a panel of DNA-inducible molecules, including MHC class II, class II transactivator (CIITA), CD40, CD80, CD86, IFN-β, TNF-α, IL-6, and CCL2 (67, 70). These results support the hypothesis that sterile cell injury can induce aberrant expression of MHC class II and costimulatory molecules and stimulate an innate immune response in the thyrocytes. On the other hand, transfected cytosolic dsDNA, but not ssDNA, suppressed iodide uptake and thyroid-specific functional gene sodium/iodide symporter (*Slc5a5*) expression in the thyrocytes (70). This result is in agreement with previous observations that thyroid function was suppressed when immune activation was induced in the thyroid (78). Furthermore, mass spectrometry analysis identified histone H2B as a thyrocyte cytosol protein that bound to a dsDNA Sepharose column (70). Knockdown of histone H2B by siRNA abolished cell injury-induced innate immune activation and increased sodium/iodide symporter (NIS) expression (70), indicating that histone H2B may serve as a cytosolic DNA sensor that mediates the immune-activating effect of DNA in the cytosol.

**Figure 2:**
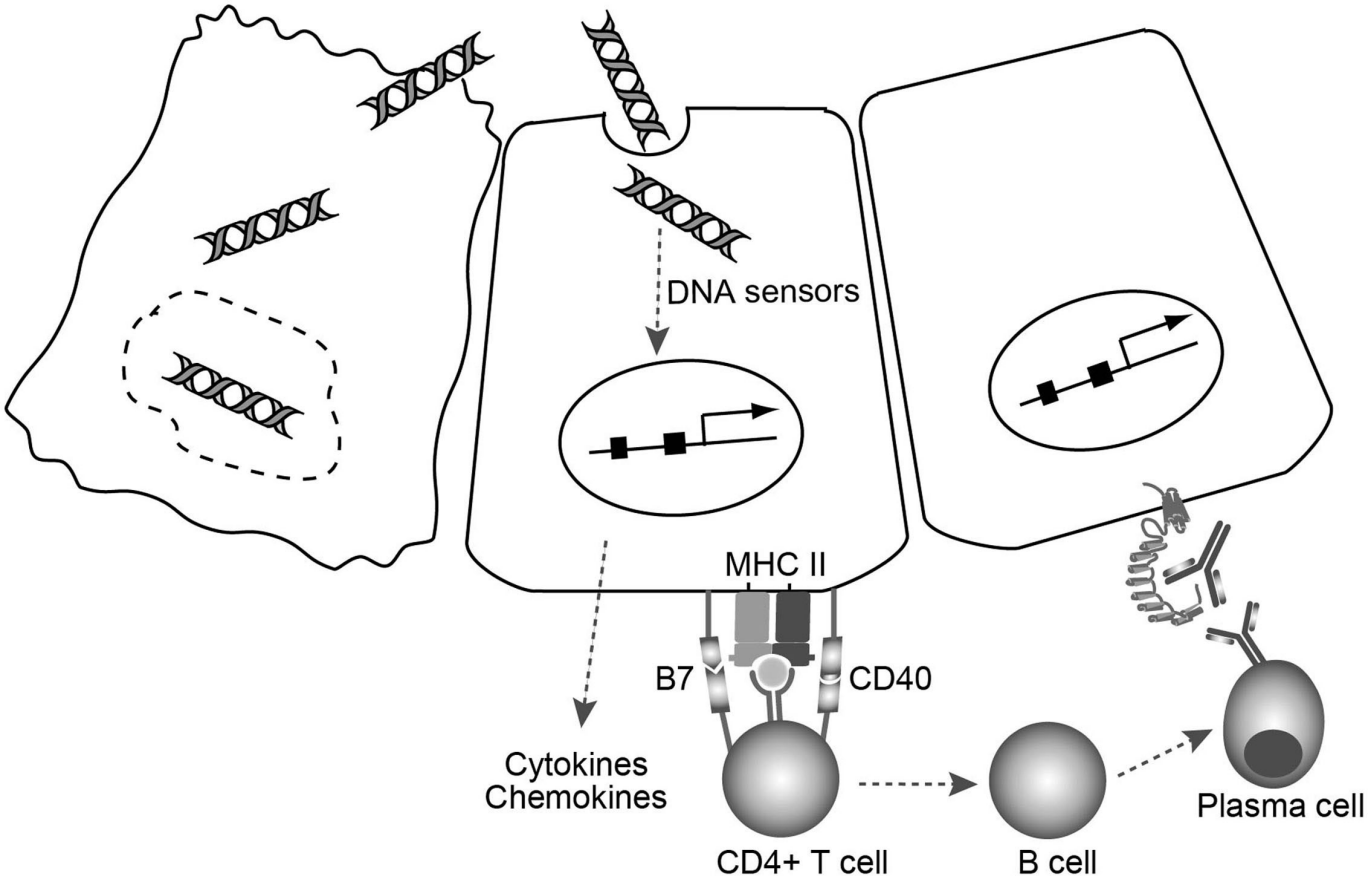
**A model for TSAb generation triggered by cell damage-derived self-genomic DNA**. Self-genomic DNA released from damaged thyrocytes (left) enters neighboring cells (middle) and induces aberrant expression of MHC class II as well as costimulatory molecules needed for the MHC class II antigen presentation pathway in thyrocytes. At the same time, cytosolic DNA stimulates thyrocytes to produce various proinflammatory cytokines and chemokines that can recruit and activate helper T cells. CD4^+^ T cells that bind to MHC class II-antigen molecule cause B cell activation. The activated B cells then differentiate into plasma cells that may produce functional TRAbs to stimulate TSHR on the thyrocytes (right).

Based on these studies, a novel model in which cell injury triggers thyroid autoimmune reactions *via* cytosolic DNA signals in the thyrocytes has been proposed (79). In this model (Figure 2), fragments of self-genomic DNA released from damaged cells may enter neighboring cells to induce expression of essential molecules in the MHC class II antigen presentation pathway, and the production of type I IFN, proinflammatory cytokines, and chemokines that can recruit and activate lymphocytes and thyrocytes. Endogenous cytosolic TSHR or internalized shed TSHR A subunit may be presented by aberrantly expressed MHC class II on the thyrocytes, with the help of costimulatory molecules to fully activate CD4^+^ cells. CD4^+^ T cells that bind to MHC class II-antigen molecules cause activation of B cells, which then differentiate into plasma cells that may produce functional TRAbs to stimulate TSHR. Thus, the cooperation of innate immune activation, inflammation, and aberrant expression of MHC class II and costimulatory molecules will consequently precipitate the generation of TSAbs under an autoimmune-prone genetic background (79).

## Conclusion

A number of successful mouse/hamster models of Graves’ disease have been established during the past two decades. Although each model has some limitations, together they have provided invaluable insight for understanding human Graves’ disease. To summarize findings from these models: (1) similar immunization approaches yielded different outcomes in different mouse strains, indicating that genetic background plays an essential role in the development of TSAb; (2) free TSHR A subunits show significant advantages over full-length TSHR for inducing TSAbs, suggesting that the epitopes recognized for the generation of functional TRAbs are likely exposed in the free TSHR A subunit; and (3) Shimojo’s model has particularly emphasized that aberrant expression of MHC class II on non-APCs is a contributing factor to Graves’ disease. In other words, the involvement of the MHC class II antigen presentation pathway in non-APCs (thyrocytes) may be an important step that leads to breaking of self-tolerance (36, 38, 80).

However, all existing animal models, strictly speaking, are not authentic Graves’ models as these animals were artificially immunized with antigens to induce antibody responses. The desired models that reflect the actual pathogenesis of Graves’ disease would have spontaneous disease onset without any artificial immunization of the causative antigen, i.e., TSHR, solely by modulating other factors that are suspected to trigger and/or accelerate autoimmune reactions. Such modulations may include the use of thyroid MHC class II/HLA-DR3 or CIITA transgenic mice, transfer of IFN-γ-pretreated syngeneic thyrocytes, increasing intramolecular cleavage and shedding rate of *in vivo* TSHR on the thyrocytes, biasing the immune balance of the extracellular milieu by cytokine/chemokine administration, raising animals with special diets or housing environment, or these conditions in combination. If such a spontaneous model was to be successfully established, even with very low disease incidence will be the true model for understanding Graves’ disease pathogenesis and even to prevent and cure Graves’ disease.

Inspired by the discovery that cytosolic DNA structures can induce aberrant MHC class II expression on various non-APCs, including thyrocytes, and stimulate the production of various immune mediators, a DNA effect may pave the way for the success of genetic vaccination approaches. The demonstration that sterile cell injury results in cytosolic DNA accumulation correlated with aberrant expression of MHC class II and costimulatory molecules, as well as inflammatory cytokines and chemokines in thyrocytes, raised the possibility that cell injury may affect self-tolerance *via* cytosolic DNA signals. From this perspective, cellular DNA is not just a genetic code but also serves to alert adjacent healthy cells to danger by interacting with a series of cellular sensors. Moreover, if the amount of cytosolic DNA that is derived from severe tissue damage and/or its deficient clearance exceeds a certain threshold, maintenance of self-tolerance may be at risk. The discovery that cell injury-derived excess self-DNA is a potential trigger for initiating thyroid autoimmune reactions may also help to generate an authentic Graves’ animal model in the future.

## Author Contributions

All the authors cooperatively conceived, analyzed, wrote, edited, and approved this review.

## Conflict of Interest Statement

The authors declare that the research was conducted in the absence of any commercial or financial relationships that could be construed as a potential conflict of interest.
